# Response to energy depletion: miR-451/AMPK loop

**DOI:** 10.18632/oncotarget.4606

**Published:** 2015-06-23

**Authors:** Agnieszka Bronisz, E. Antonio Chiocca, Jakub Godlewski

**Affiliations:** Department of Neurosurgery, Harvey Cushing Neuro-oncology Laboratories, Brigham and Women's Hospital, Harvard Medical School, Boston, MA, USA

The adaptation of cancer cells to the constantly changing conditions of their microenvironment during tumor progression requires dynamic and flexible mechanisms. Glioblastoma and other tumors cells require a continuous flux of nutrients and oxygen to sustain growth and elevated metabolism; yet, there is often insufficient supply of blood and energy to nourish this growth/metabolism. While the adaptive mechanisms to reduced oxygen (hypoxia) have been well defined, the adaptations to fluctuations in the major energy nutrient– i.e. glucose, are poorly comprehended. It is likely that the successful survival of a cancer cell depends on its ability to frequently and dynamically adjust for nutrient fluctuations.

Reprogrammed glucose metabolism as a result of increased glycolysis and glucose uptake is a hallmark of numerous solid tumors and it was recently demonstrated that enhancing glucose uptake is one of the mechanisms of adaptation of glioblastoma cells to limited glucose availability [1]. The preferred uptake of glucose by cancer cells led us to hypothesize that in addition to an intracellular “sensor” pathway that monitors the fluctuations of environmental glucose; there must be the effector mechanisms that mediate adaptative response.

It is known that cells exposed to low glucose become metabolically stressed. This results in a shortage of ATP, increasing the [AMP]/[ATP] ratio that activates the 5′AMP-activated protein kinase (AMPK) complex. AMPK is a highly conserved energy sensor belonging to a class of serine/threonine kinases that controls cell metabolism during environmental stress. When cellular energy levels are decreased (and thus the AMP/ATP ratio is increased), AMPK is phosphorylated by LKB1 [2]. Although the role of LKB1/AMPK axis in metabolic homeostasis is well documented, its function in cancer is much less clear. Our group has shown that the non-coding microRNA – miR-451, is a potent inhibitor of the AMPK signaling pathway [3] directly targeting CAB39 - a necessary LKB1 co-activator. Glucose availability modulated the expression of miR-451 in glioblastoma cells. High glucose led to high levels of miR-451, shutting-off AMPK function, inhibiting cell migration and elevating cell proliferation. Conversely, low glucose led to AMPK activation, which diminished the levels of miR-451, inhibited cell growth and turned on a migratory phenotype [3]. These observations led us to postulate the existence of an AMPK/miR-451 reciprocal negative feedback loop, mediated by glucose availability. However, the molecular effectors facilitating the low glucose/active AMPK-mediated drop in miR-451 levels were not known.

In our recent study, we showed that indeed the miR-451/AMPK loop is transcriptionally regulated [4]. Regardless of the tested experimental glucose regimen (withdrawal, gradual depletion, or surge replenishment), the levels of the primary transcript of miR-451 were dynamically and reversibly linked to glucose status. The analysis of the region upstream to the miR-451 genomic locus revealed multiple putative binding sites for OCT1–a widely expressed transcription factor [5]. We demonstrated that the dynamic binding of OCT1 to its predicted binding sites located 6–8 kb upstream of the miR-451 locus led to the recruitment of RNA polymerase II and initiated the transcription of miR-451 gene. OCT1 transcriptional activity (i.e. the ability to bind DNA) is inhibited by the phosphorylation at Ser 335 [5]. In low glucose conditions, active AMPK directly phosphorylates OCT1 at Ser 335, preventing its function. This mechanism thus links glucose availability to OCT1's transcriptional function on the miR-451 promoter.

Oct1-deficient cells are resistant to glucose deprivation due to a reduction of glucose metabolism [6] and are characterized by exceptionally low levels of miR-451. Conversely, in AMPK-deficient cells, OCT1 remained largely dephosphorylated in low glucose, resulting in the expression of miR-451 [4]. These results thus show that the AMPK/OCT1/miR-451/LKB1 loop provides a nutrient-dependent regulatory mechanism to allow the cell to adapt to changing microenvironmental conditions.

Because AMPK can impede cell growth, it was historically perceived as a *bona fide* tumor suppressor. However, recently, a number of studies have emerged that lead to the opposite conclusion, namely that the AMPK complex endows cancer cells with the ability to survive “stressors”, including energy and growth factor deficiency and genomic damage [7]. It provides a potent regulatory mechanism by which cancer cells are capable of temporarily halt their growth as they face microenvironmental and therapy-inflicted challenges. Thus, AMPK can be perceived as a contextual oncogene, enabling cancer cells with behavioral and biochemical flexibility.

Both, energy-conserving metabolic shift and resource-seeking behavioral change require brain tumor cell to shut down miR-451, while forced expression of miR-451 during stress leads to cytotoxicity [3]. The AMPK-dependent inactivation of the OCT1 transcriptional activator of miR-451 helps brain tumor cells to escape from metabolically stressful events/locations. MiR-451 thus provides an example of a molecule that is not deregulated in brain tumor cells, but is instead finely regulated by promoting or suppressing brain tumor cell phenotypes based on microenvironmental contexts.
